# Novel arabinan and galactan oligosaccharides from dicotyledonous plants

**DOI:** 10.3389/fchem.2014.00100

**Published:** 2014-11-10

**Authors:** Daniel Wefers, Catrin E. Tyl, Mirko Bunzel

**Affiliations:** ^1^Department of Food Chemistry and Phytochemistry, Institute of Applied Biosciences, Karlsruhe Institute of TechnologyKarlsruhe, Germany; ^2^Department of Food Science and Nutrition, University of MinnesotaSt. Paul, MN, USA

**Keywords:** oligosaccharides, NMR, cell wall, polysaccharides, pectin, arabinans, galactans, ferulic acid

## Abstract

Arabinans and galactans are neutral pectic side chains and an important part of the cell walls of dicotyledonous plants. To get a detailed insight into their fine structure, various oligosaccharides were isolated from quinoa, potato galactan, and sugar beet pulp after enzymatic treatment. LC-MS^2^ and one- and two-dimensional NMR spectroscopy were used for unambiguous structural characterization. It was demonstrated that arabinans contain β-(1→3)-linked arabinobiose as a side chain in quinoa seeds, while potato galactan was comprised of β-(1→4)-linked galactopyranoses which are interspersed with α-(1→4)-linked arabinopyranoses. Additionally, an oligosaccharide with two adjacent arabinofuranose units *O*2-substituted with two ferulic acid monomers was characterized. The isolated oligosaccharides gave further insight into the structures of pectic side chains and may have an impact on plant physiology and dietary fiber fermentation.

## Introduction

Pectins are among the main cell wall polysaccharides of dicotyledonous plants. Arabinans and galactans are neutral side chains of rhamnogalacturonan-I, which is part of the pectic polysaccharides. Neutral pectic side chains are often a major part of the cell wall polysaccharides in dicotyledonous plants and involved into important physiological processes in the plant, for example fruit softening (Pena and Carpita, 2004). The main structural characteristics of arabinans and galactans are well known. They are attached to the rhamnogalacturonan-I backbone at position *O*4 of the rhamnosyl residues. Arabinans are composed of a backbone of α-(1→5)-linked arabinofuranoses, which may be branched at position *O*3 and/or *O*2. Galactans are comprised of a β-(1→4)-linked backbone of galactopyranoses, which can be decorated by terminal arabinofuranoses and galactopyranoses (Voragen et al., 2009). In plants of the order of Caryophyllales, ferulic acid can be attached to the *O*2-position of the arabinoses and the *O*6-position of the galactoses, adding additional complexity to pectin structures (Ishii and Tobita, 1993; Colquhoun et al., 1994; Bunzel et al., 2005). Ferulic acid substitution may result in cross-linking of polysaccharide chains due to oxidative dimerization of the ferulates, altering the physicochemical properties of these polymers, the cell walls of plants, and plant based food products (Bunzel et al., 2001). Also, ferulic acid can be liberated by the human microbiota in the large intestine (Holck et al., 2011). Liberated ferulic acid and its microbial metabolites are potentially reabsorbed from the colon (Zhao and Moghadasian, 2010). Arabinans and galactans also are part of the dietary fiber complex and are suggested to have prebiotic effects (Van Laere et al., 2000; Thomassen et al., 2011). Most often, the main structural features of pectic side chains are determined only. Despite having a major impact on physiological effects, more complex structural elements are often not analyzed or cannot be determined by routine methods.

In this study, we isolated three oligosaccharides from different dicotyledonous plants, representative of pectin structural features. They were characterized by LC-MS^2^ and one- and two-dimensional NMR spectroscopy. Novel structural features were demonstrated, broadening our knowledge about the structures of neutral pectic side chains.

## Materials and methods

### Plant materials and sample preparation

Quinoa seeds (*Chenopodium quinoa* Willd.), grown and harvested 2012 in Bolivia, were from a local supplier. They were milled to a size < 0.5 mm, defatted with acetone, and used for fiber preparation. Insoluble dietary fiber was isolated before enzymatic digestion of the non-starch polysaccharides. Starch was degraded by incubating 20 g of quinoa flour, suspended in 200 mL of phosphate buffer (pH 6.2), with thermostable α-amylase (1.5 mL, Termamyl 120L, EC 3.2.1.1, from *B. licheniformis*, 120 KNU/g, kindly donated by Novozymes, Bagsvaerd, Denmark) for 20 min at 95°C. After adjusting the pH to 7.5 with sodium hydroxide, 1.12 g protease (Alcalase 1.5 MG Type FG, EC 3.4.21.62, from *B. licheniformis*, 1.5 AU/g, kindly donated by Novozymes) were added and the mixture was incubated for 30 min at 60°C. The pH was adjusted to 4.5 with hydrochloric acid, and amyloglucosidase was added (700 μL, AMG 300L, EC 3.2.1.3, from *A. niger*, 300 AGU/g, kindly donated by Novozymes). After incubation for 30 min at 60°C, the mixture was filtrated, and insoluble fiber was washed with water, ethanol (99%), and acetone.

Insoluble fiber (5 g) was suspended in 500 mL of bidistilled water and 50 U endo-arabinanase (9 U/mg, EC 3.2.1.99, from *A. niger*, Megazyme, Bray, Ireland) were added. The mixture was incubated for 24 h, heated to 95°C for 5 min and centrifuged to remove the undigested residue. The sample was freeze-dried before further fractionation.

Pectic galactan from potato (*Solanum tuberosum* L.) was from Megazyme. The galactan (200 mg) was dissolved in 20 mL of bidistilled water and incubated with 25 mg of Driselase^®^ (Sigma Aldrich, Schnelldorf, Germany) for 24 h at 40°C. Undigested polysaccharides and higher oligosaccharides were precipitated with ethanol (99%)/water (80/20, v/v) and removed by centrifugation. After evaporation and lyophilization, charged rhamnogalacturonan-I oligosaccharides were removed by passing the redissolved hydrolyzate through an LC-SAX SPE column (Supelco, Sigma Aldrich).

Sugar beet pulp (*Beta vulgaris* L. subsp. *vulgaris*), kindly provided by Suedzucker (Mannheim, Germany), was freeze-dried and milled to a size < 0.5 mm. Milled pulp (< 0.5 mm, 80 g) was suspended in 5 L of bidistilled water, and 8 g of Viscozyme L (Novozymes) were added. The mixture was incubated for 48 h at 40°C, followed by heating to 95°C for 5 min. The hydrolyzate was loaded onto a column (45 × 5 cm) packed with Amberlite XAD-2 (Supelco, Sigma Aldrich). The column was washed with 1 L of water, the phenolic acid oligosaccharides were eluted with 750 mL of methanol, and the eluate was evaporated to 10 mL (sample loop size at Sephadex LH-20 chromatography).

### Oligosaccharide isolation

The isolation procedures for compound 1 and 2 were very similar. The freeze-dried oligosaccharides were redissolved in bidistilled water (10 mL) and fractionated by using Bio-Gel P2 chromatography (Bio-Rad Laboratories, Hercules, CA, USA) (sample loop size: 10 mL, bed volume: 85 × 2.6 cm). Elution was carried out with bidistilled water (1 mL/min) at 45°C, and the effluent was monitored by refractive index detection (Smartline RI detector 2300, Knauer, Berlin, Germany). Collected fractions were freeze-dried, and further fractionation was performed on an HPLC system (L-7100 pump, L-7490 RI detector, Merck/Hitachi, Darmstadt, Germany) equipped with a semipreparative C18 column (250 × 8 mm, 5 μm, Eurosphere 100, Knauer). Bidistilled water was used as eluent (1.5 mL/min for compound 1, 1 mL/min for compound 2). Separations were performed at 5°C (compound 2) and 25°C (compound 1).

For isolation of compound 3, the hydrolyzate was loaded onto a Sephadex LH-20 column (85 × 2.6 cm, GE Healthcare Biosciences, Pittsburgh, PA, USA). Elution was carried out with water (0.5 mL/min), water/methanol (70/30, v/v), and water/methanol (30/70, v/v, 1 mL/min; UV detection at 325 nm). The collected fractions were concentrated and purified by semipreparative HPLC (AZURA P2.1L pumps, UVD2.1L UV detector, Knauer) using a semipreparative Luna C18 column (250 × 10 mm i.d., 5 μm particle size, Phenomenex, Torrance, CA, USA). The following gradient composed of water (A) and methanol (B) was applied: 0–2.5 min 20% B; 2.5–30 min from 20% B to 100% B; 30–45 min 100% B; 45–46 from 100% B to 20% B; 46–50 min 20% B. A flow rate of 2 mL/min was used, and UV-detection was carried out at 325 nm.

### Monosaccharide analysis

An aliquot of the oligosaccharides was hydrolyzed with 2 M trifluoroacetic acid (TFA) at 121°C for 30 min. After evaporation, the samples were redissolved in water and analyzed by HPAEC-PAD on an ICS-5000 System (Thermo Scientific Dionex, Sunnyvale, CA, USA) using a CarboPac PA-20 column at 25°C. A flow rate of 0.4 mL/min and the following gradient composed of (A) bidistilled water, (B) 0.1 M sodium hydroxide, (C) 0.1 M sodium hydroxide + 0.2 M sodium acetate were used: Before every run, the column was rinsed with 100% B for 10 min and equilibrated for 20 min with 90% A and 10% B. After injection, the following gradient was applied: 0–1.5 min, from 90% A and 10% B to 96% A and 4% B; 1.5–22 min, isocratic, 96% A and 4% B; 22–32 min from 96% A and 4% B to 100% B; 32–42 min, isocratic, 100% C.

To determine the d/l-monosaccharide configuration, the evaporated TFA hydrolyzate was heated overnight at 130°C with 150 μL of (R)-2-octanol and 5 μL of TFA as described by Leontein et al. (1978). Solvent was removed and samples were silylated by using 80 μL of *N,O*-bis(trimethylsilyl)trifluoroacetamide and 20 μL of pyridine. The samples were analyzed by GC-MS (GC-2010 Plus and GCMS-QP2010 Ultra, Shimadzu, Kyoto, Japan) equipped with an Rxi-5Sil MS column (30 m × 0.25 mm i.d., 0.25 μm film thickness, Restek, Bad Homburg, Germany) using the following conditions: initial column temperature, 150°C; ramped at 1°C/min to 200°C; ramped at 15°C/min to 300°C. Split injection was performed at a split ratio of 1:10, the injection temperature was 275°C. Helium was used as carrier gas at 40 cm/s, and the transfer line was held at 275°C.

### LC-MS^2^ characterization

LC-MS^2^ was carried out on a Surveyor HPLC System, coupled to an LXQ linear ion trap MS^n^ system (Thermo Fisher Scientific, Waltham, MA, USA). For analysis of compound 1 and 2, a porous graphitized carbon (PGC) column (Hypercarb, 100 × 2.1 mm, 3 μm) (Thermo Fisher Scientific) was used. The following gradient composed of 25 μM aqueous LiCl (A) and acetonitrile (ACN) (B) was used: 0–1 min, 100% A; 1–20 min, from 100% A to 90% A; 20–28 min, from 90% A to 30% A; 28–31 min, from 30% A to 20% A; 31–35 min, isocratic 20% A; 35–36 min, from 20% A to 100% A; 36–41 min, equilibration with 100% A. A flow rate of 0.4 mL/min was applied, and the column was heated to 60°C.

Due to weak ionization of compound 3, this compound was injected directly into the ESI source (negative mode) by using a syringe pump at 20 μL/min.

### NMR characterization

The oligosaccharides were hydrogen-deuterium exchanged and dissolved in D_2_O (compound 1 and 2) or acetone/D_2_O 3:1 (compound 3). Spectra from ^1^H, H,H-Correlated Spectroscopy (COSY), Heteronuclear Multiple-Quantum Correlation (HMQC), and Heteronuclear Multiple Bond Correlation (HMBC) experiments were acquired on a Bruker (Rheinstetten, Germany) Ultrashield 700 MHz NMR. Internal acetone was used as an internal reference [2.22/30.89 ppm for compound 1 and 2 (Gottlieb et al., 1997), 2.04/29.80 ppm for compound 3 (Allerdings et al., 2005)].

## Results

### Compound 1

**Figure 1:**
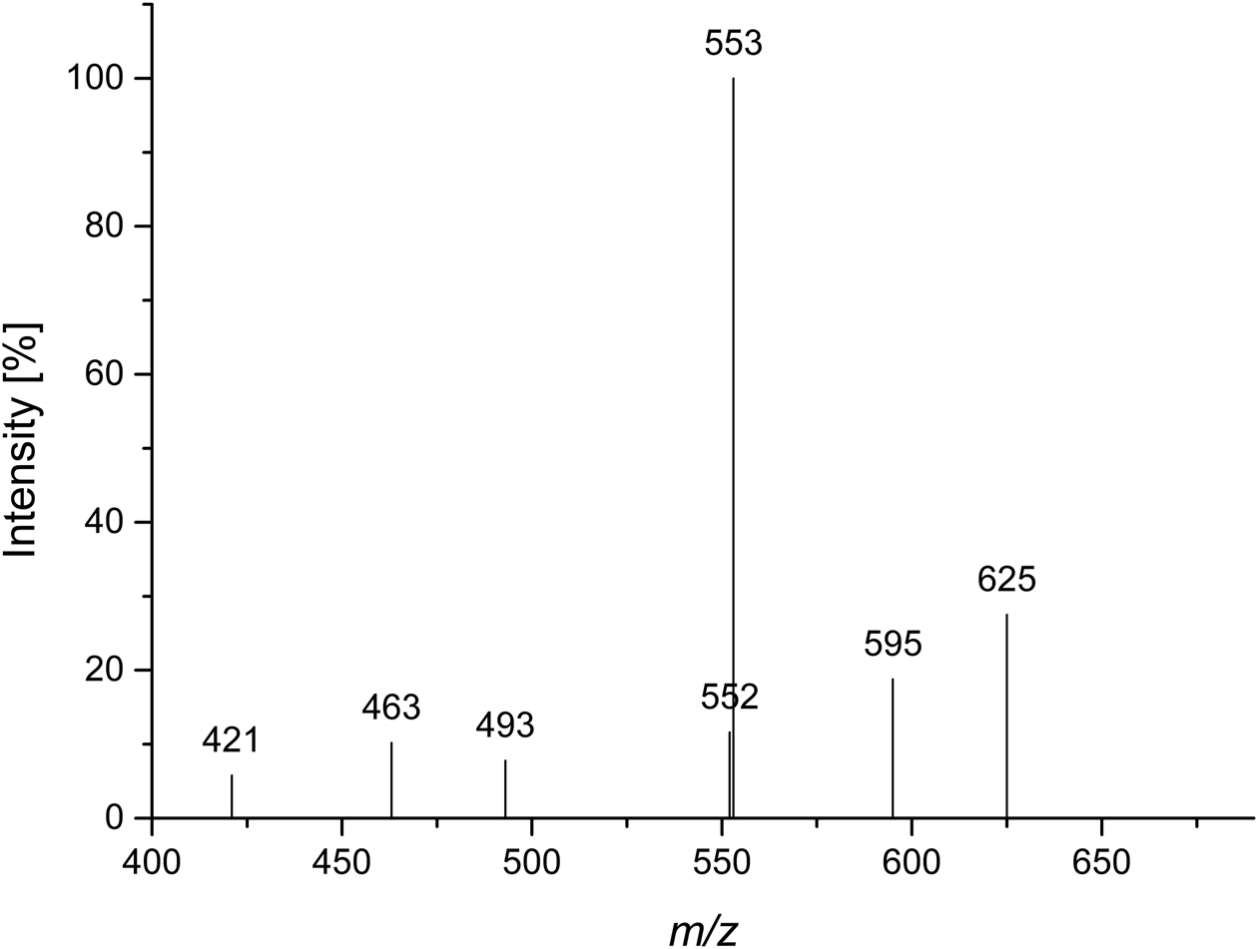
**CID MS^2^ mass spectrum of lithium cationized compound 1 (*m/z* 685)**.

Quinoa insoluble fiber was hydrolyzed with *endo*-arabinanase, and the hydrolyzate was fractionated using Bio-Gel P2 chomatography. Semipreparative HPLC purification of the corresponding fractions yielded compound 1 in sufficient amounts and purity for further analysis. Monosaccharide analysis showed that l-arabinose was the only monosaccharide present. LC-PGC-MS of the lithium-cationized oligosaccharide showed a quasimolecular ion with *m/z* 685, indicating an arabinose pentamer. Since *endo*-arabinanase should be able to cleave a linear pentamer, the isolated pentamer was hypothesized to be branched. This hypothesis was supported by the MS^2^ spectrum (Figure 1). Westphal et al. (2010b) showed that cross ring cleavages are the source of the main fragments of linear arabinooligosaccharides, while MS^2^ spectra of branched oligosaccharides show higher intensities from interglycosidic cleavages. The dominating fragment ion is *m/z* 553 (mass loss of 132 Da), which corresponds to the loss of an anhydro-arabinose. The 60 Da (*m/z* 625) and 90 Da (*m/z* 595) mass losses result from cross ring cleavage through the reducing unit. Because these fragments are of a much lower intensity than the interglycosidic cleavage fragment, compound 1 seems to be a branched arabinan pentamer.

**Figure 2:**
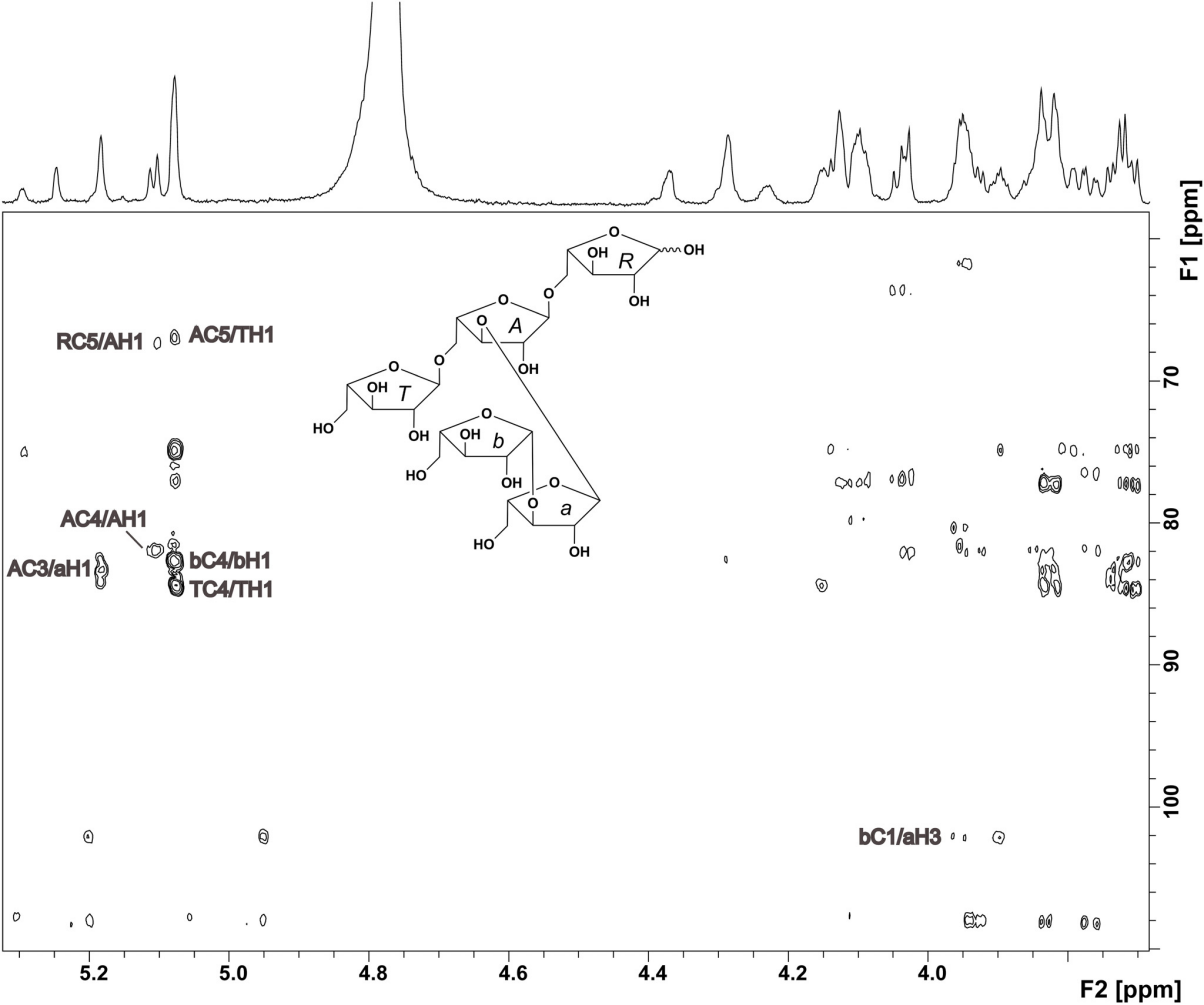
**HMBC spectrum and structure of compound 1**. Diagnostic long range correlations are marked and explained in the text.

The oligosaccharide showed a complex ^1^H NMR spectrum, requiring 2D NMR experiments for unambiguous structure elucidation. A COSY spectrum allowed for the assignment of the corresponding ring protons to the well resolved anomeric protons. The signal at 5.08 ppm represents two H1 protons as indicated by the HMQC spectrum. The ^13^C chemical shifts of units *R*, *A*, and *T* (Table 1), which were obtained from the HMQC spectrum, were in good agreement with literature data for a reducing (*O*5 substituted), an α-(1,3,5)-substituted, and a terminal α-arabinofuranose (Westphal et al., 2010a). This was confirmed by HMBC cross peaks between C5 of unit *A* and H1 of unit *T*, and between C5 of unit *R* and H1 of unit *A* (Figure 2). Additionally, there were cross peaks between C4 and H1 for unit *A* and *T*, confirming the furanose form. The *O*3 substitution of unit *A* was confirmed by a cross peak between H1 of unit *a* and C3 of unit *A*. The ^13^C chemical shifts of unit *a* suggested an α-linked arabinose in furanose form. The δ_C5_ value of unit *a* is similar to the δ_C5_ value of unit *T*. Also, the carbon signal of C3 of unit *a* is shifted downfield to 84.48 ppm. This suggests that this arabinose is substituted at position *O*3 instead of *O*5. The ^13^C chemical shifts of the remaining arabinose unit *b* are different from those of the other arabinoses, but comparable with data for a terminal β-linked arabinofuranose (Cardoso et al., 2002). The furanose form was confirmed by the corresponding HMBC cross peak between C4 and H1. A weak HMBC cross peak between C1 of unit *b* and H3 of unit *a* (Figure 2) suggests that unit *b* is linked to position *O*3 of unit *a*. In conclusion, NMR and MS data suggest the structure shown in Figure 2 for compound 1.

**Table 1:** **^1^H and ^13^C chemical shifts and coupling constants of the isolated oligosaccharides**.

**COMPOUND 1**					
**Unit**	**1**	**2**	**3**	**4**	**5**
R-α	5.25	4.03	4.04	4.23	3.76/3.83
	101.92	82.07	76.60	82.04	67.35
R-β	5.30	4.10	4.09	3.94	3.76/3.83
	96.18	76.80	75.04	79.92	67.35
A	5.10/5.11	4.29	4.11	4.29	3.85/3.95
	108.19	79.94	82.69	82.05	67.02
T	5.09	4.13	3.95	4.10	3.82/3.70
	108.15	81.74	77.26	84.69	61.86
a	5.19	4.37	3.96	4.16	3.72/3.84
	107.87	80.38	84.48	83.43	61.79
b	5.08	4.12	4.04	3.90	3.72/3.82
	102.26	77.02	74.95	82.73	63.78

**Table:** 

**COMPOUND 2**						
**Unit**	**1**	**2**	**3**	**4**	**5**	**6**
R-α	5.27 (4.0)	3.86	3.96	4.23	4.12	3.72/3.83
	93.04	69.23	70.40	79.49	70.47	61.62
R-β	4.60 (8.0)	3.58	3.76	4.17	3.76	3.72/3.83
	97.17	72.92	74.14	78.47	74.14	61.62
A	4.64 (8.0)	3.66	3.78	4.14	3.71	3.79
	105.12	72.75	74.12	78.17	75.48	61.61
T	4.57 (8.0)	3.62	3.65	69.25	3.68	3.77/3.79
	105.47	71.77	73.09	3.91	75.86	61.59
a	4.60 (8.0)	3.68	3.73	4.08	3.64/4.21	–
	105.22	72.58	73.58	78.92	66.72

**Table:** 

**COMPOUND 3**										
**Unit**	**1**	**2**	**3**	**4**	**5**	**6**	**7**	**8**	**9**	**10**
R-α	5.13 (2.3)	3.93	3.91	4.13	3.62/3.80	–	–	–	–	–
	102.38	82.64	77.31	82.01	67.95					
R-β	5.17 (4.4)	3.93	3.91	3.82	3.62/3.80	–	–	–	–	–
	96.69	75.86	77.31	80.58	67.95					
A	5.06/5.05	5.02	4.20	4.21	3.73/3.87	–	–	–	–	–
	106.37	84.31	76.14	83.64	66.53					
T	5.11	5.07	4.06	4.08	3.63/3.75	–	–	–	–	–
	106.45	84.57	76.17	84.84	61.72					
F_A_	–	7.20	–	n.d.	6.77 (8.0)	7.07 (8.0)	7.59 (16.0)	6.33 (16.0)	–	3.80
	n.d.	111.56	149.02	n.d.	116.29	124.26	147.30	113.49	n.d.	55.97
F_B_	–	7.12	–	n.d.	6.77 (8.0)	6.99 (8.0)	7.54 (16.0)	6.27 (16.0)	–	3.78
	n.d.	111.40	149.00	n.d.	116.32	124.13	147.32	113.42	n.d.	55.97

The corresponding structures including the descriptors are shown in Figures 2, 4, 6. Chemical shifts are given in ppm, coupling constants in Hz. n.d., not determined.

### Compound 2

**Figure 3:**
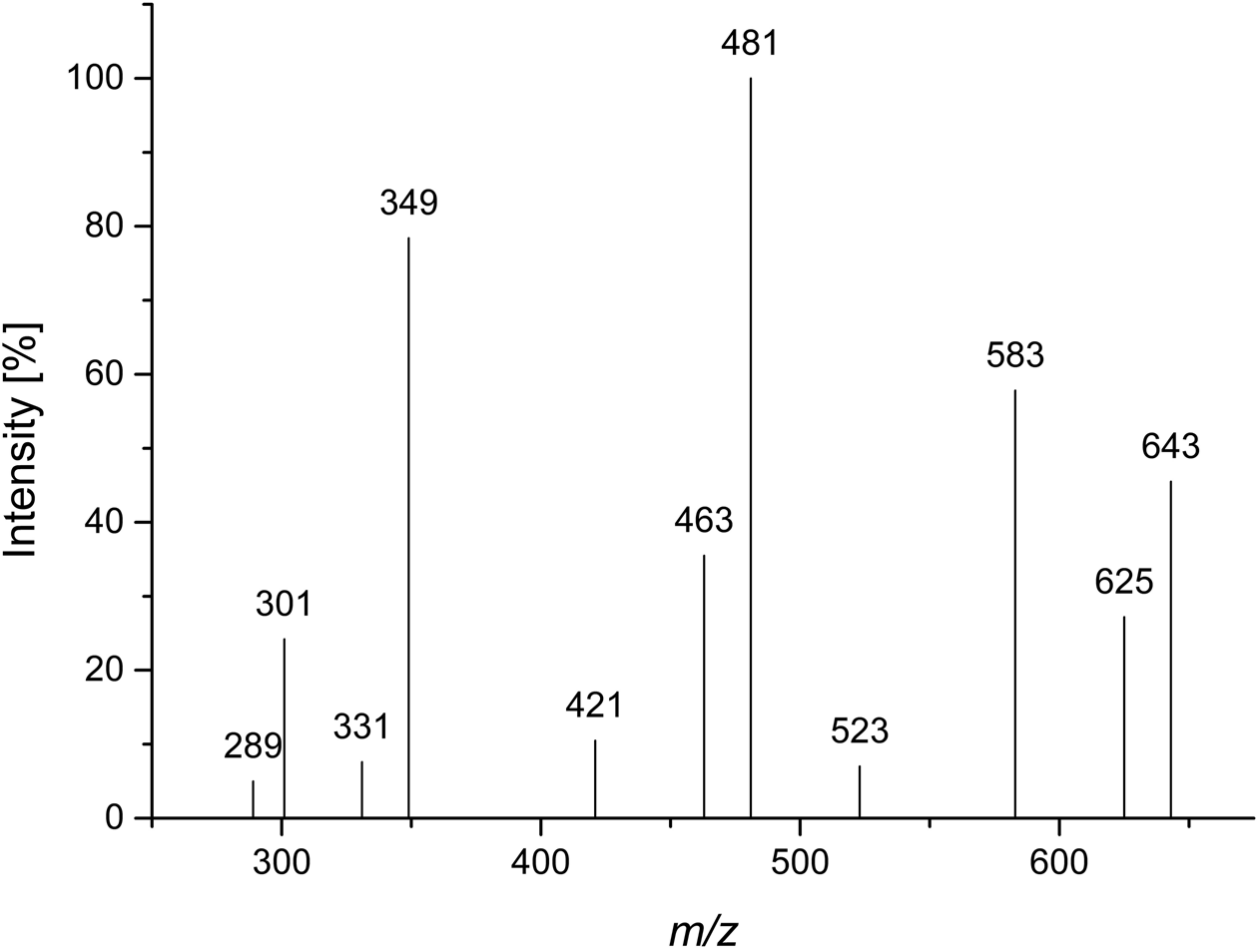
**CID MS^2^ mass spectrum of lithium cationized compound 2 (*m/z* 643)**.

Compound 2 was isolated by Bio-Gel P2 chromatography and semipreparative HPLC after Driselase digestion of pectic galactans from potato. A galactose/arabinose ratio of approximately 3:1 was determined by HPAEC monosaccharide analysis. Determination of the d/l monosaccharide configuration by GC-MS showed that arabinose was present in its l-configuration and galactose in its d-configuration. LC-PGC-MS resulted in a very broad peak with *m/z* 643 (corresponding to the lithium adduct of an oligosaccharide made up of three galactoses and one arabinose). The peak broadening is typical for sugars in their pyranose form on reversed phase columns. Besides the quasimolecular ion (*m/z* 643), the MS^2^ fragmentation pattern showed a high intensity of a 60 Da mass loss (*m/z* 583) and a water loss (*m/z* 625) (Figure 3). Hofmeister et al. (1991) investigated MS^2^ fragmentation patterns of lithium cationized hexopyranose disaccharides and described the mass losses observed here as characteristic for an (1→4)-linkage. The main fragment at *m/z* 481 represents the loss of anhydro-galactose. The absence of fragments representing the loss of an arabinose residue (mass losses of 132 Da or 150 Da) suggests that arabinose is not present in a terminal position. The fragments at *m/z* 463 and at *m/z* 421, which could represent 18 Da and 60 Da mass losses of *m/z* 481, may point to a (1→4)-linkage of the internal galactose. The intense fragment at *m/z* 349 may result from a 132 Da mass loss of *m/z* 481 or of a combined loss of galactose and arabinose. Although not all fragments can be explained, LC-MS^2^ points to an (1→4)-linked galactan containing an internal arabinose unit.

**Figure 4:**
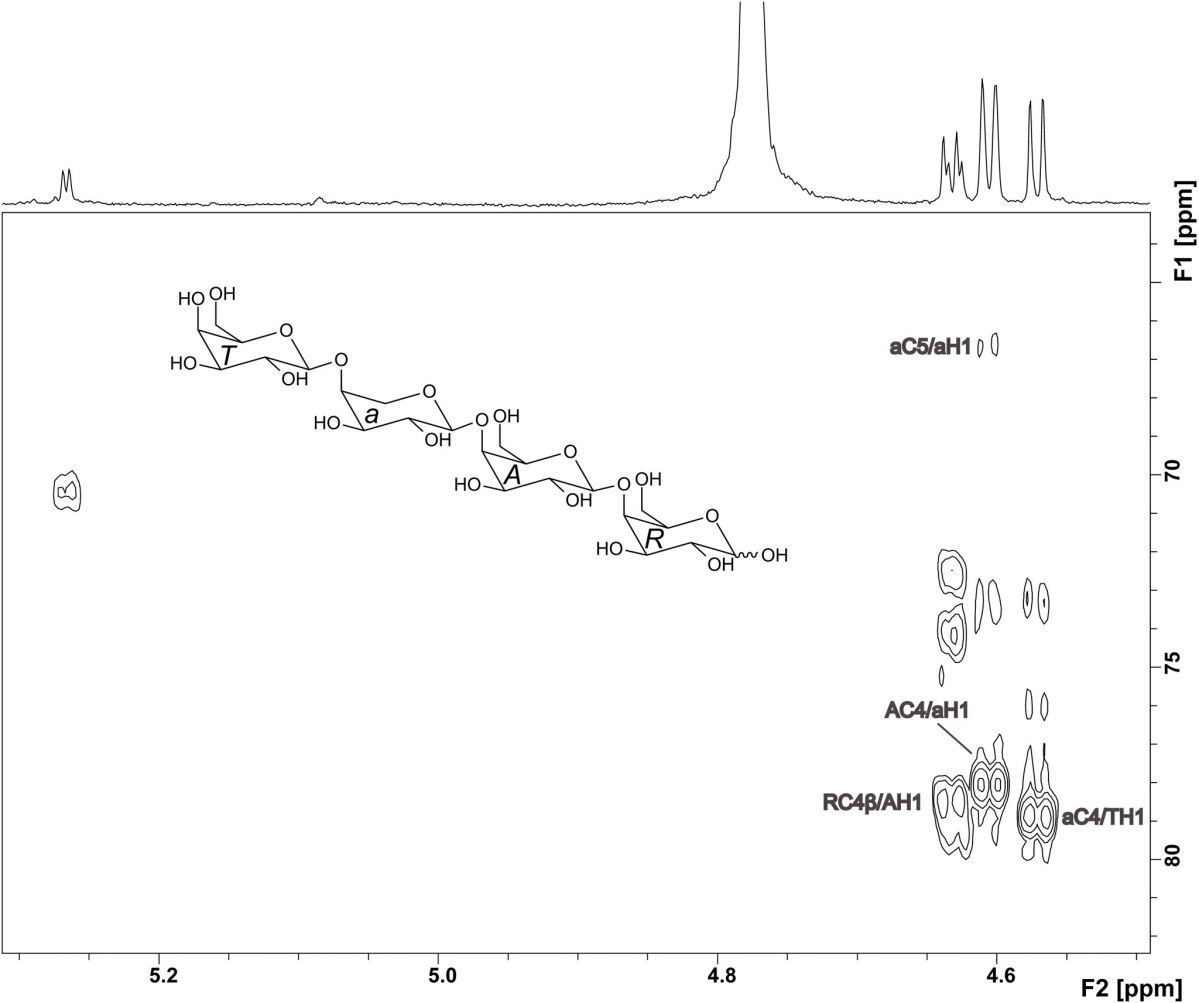
**HMBC spectrum and structure of compound 2**. Diagnostic long range correlations are marked and explained in the text.

The ^1^H NMR spectrum of compound 2 showed well resolved signals for the anomeric sugar protons between 4.5 and 4.7 ppm with coupling constants around 8 Hz, in addition to one signal at 5.27 ppm with a coupling constant of 4 Hz (Figure 4). This suggests the presence of β-linked galactopyranoses and the absence of arabinose in its furanose form (Agrawal, 1992). This hypothesis was confirmed by the HMQC spectrum. ^1^H and ^13^C chemical shifts for unit *R*, *A*, and *T* (Table 1) were comparable to those reported for a reducing 4-linked galactopyranose, a β-(1→4)-substituted galactopyranose, and a terminal β-galactopyranose (Fransen et al., 1998; Lichtenthaler et al., 2001; Ishii et al., 2005). ^13^C chemical shifts of unit *a* were in good agreement with data for an α-arabinopyranose, except that C4 is shifted downfield (Ishii et al., 2005). This suggests substitution at this position and is in good agreement with the MS^2^ experiments. An HMBC cross peak between C4 of unit *a* and H1 of unit *T* confirmed this assignment (Figure 4). The unusual ringform of the arabinose is also evidenced by a weak cross peak between C5 and H1. Additionally, cross peaks between C4 of the units *A* and *R* and H1 of the units *a* and *A* demonstrated the β-(1→4)-linkages between the units *a*, *A* and *R* (Figure 4). Thus, these data demonstrate the structure of compound 2 as shown in Figure 4.

### Compound 3

**Figure 5:**
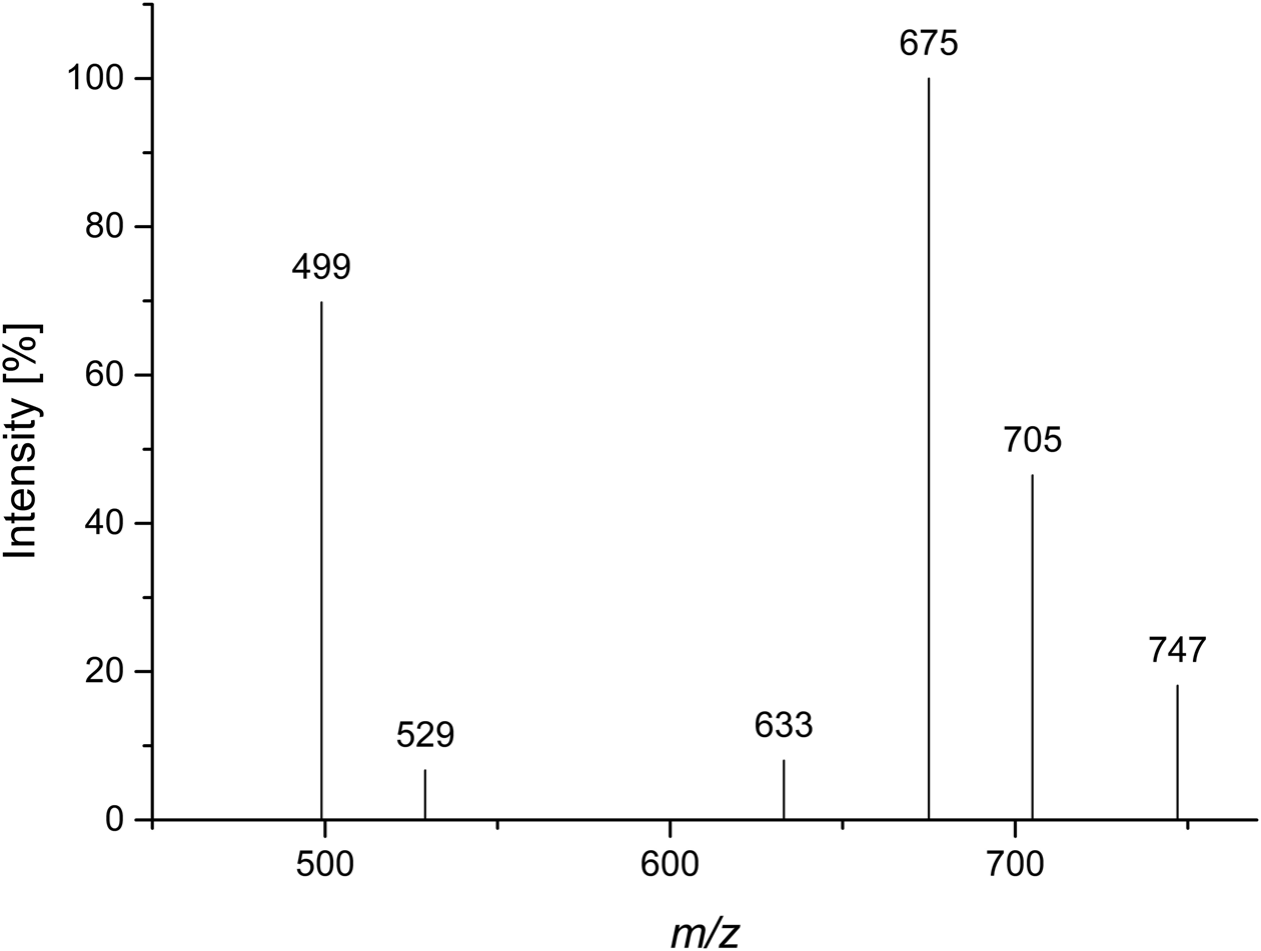
**CID MS^2^ mass spectrum of compound 3 in negative mode (*m/z* 765)**.

A Viscozyme L hydrolyzate of sugar beet pulp was fractionated by using Sephadex LH-20 chromatography, and compound 3 was obtained from the methanol/water 70/30 eluate. After semipreparative HPLC purification, monosaccharide analysis of the purified compound showed l-arabinose as the only monosaccharide present. ESI-MS showed a quasimolecular ion [M-1]^−^ at *m/z* 765, corresponding to three arabinose units, esterified with two ferulic acids. The MS^2^ spectrum yielded mass losses of 60 and 90 Da (*m/z* 705 and *m/z* 675), typical for cross ring cleavages through a 5-substituted, reducing arabinose (Figure 5). A weak interglycosidic cleavage fragment (*m/z* 633) is also present, suggesting that ferulic acid is not attached to the reducing arabinose. The remaining fragments (*m/z* 499 and *m/z* 529) may result from a mass loss of 176 Da of the fragments at *m/z* 705 and *m/z* 675, corresponding to a loss of an anhydro-ferulic acid. Levigne et al. (2004) isolated a diferuloylated oligosaccharide showing the same quasimolecular ion from sugar beet. The MS^2^ spectrum of that compound showed different intensities of the fragment ions than the compound analyzed here. It also contained only minor amounts of the fragments at *m/z* 499 and *m/z* 529. These fragments were assigned to impurities by the authors. They proposed a structure where an (1→5)-linked arabinotriose is esterified by two ferulic acids at *O*2 of the (1→5)-linked arabinose and at *O*5 of the terminal arabinose. The differences in the MS^2^ spectrum suggest that the positions of esterification could be different in the compound analyzed here.

**Figure 6:**
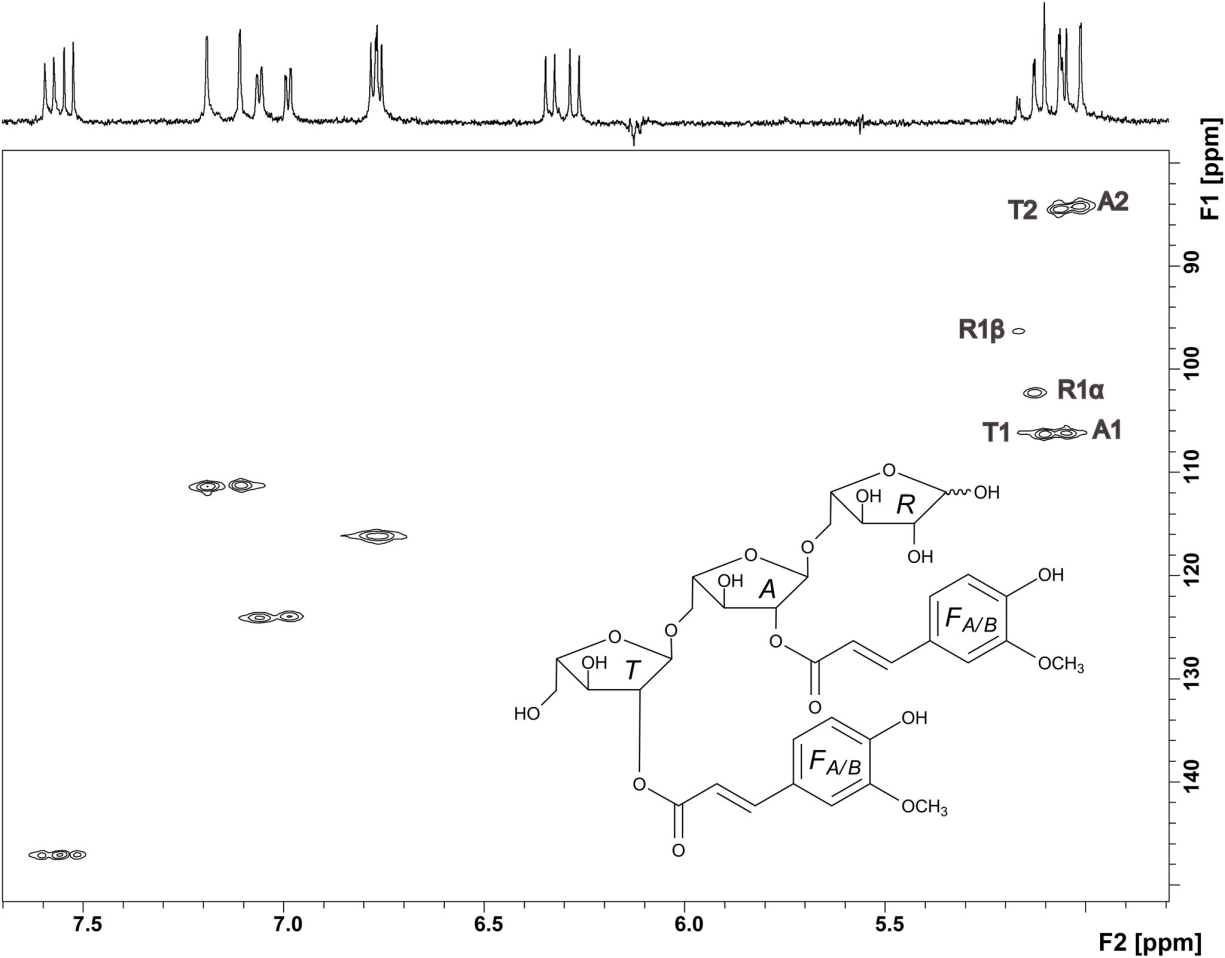
**HMQC spectrum and structure of compound 3**. Signals in the anomeric region are assigned and explained in the text.

Thus, NMR spectroscopy was required to unambiguously assign the structure of compound 3. The ^1^H spectrum confirmed the presence of two ferulic acid residues (Figure 6). Two sets of H7/H8 protons with coupling constants of 16 Hz demonstrated that both ferulic acids are present in their *trans*-configuration. The ^13^C chemical shifts are very similar for both ferulic acids and in good agreement with data for ester-linked ferulic acid (Colquhoun et al., 1994; Bunzel et al., 2005). Since the phenolic protons do not couple with the propenylic proton H7 in the COSY spectrum and because both ferulic acid units showed similar ^13^C chemical shifts (Table 1), assignments were made assuming that the downfield shifted H7 and H8 protons belong to the same ferulic acid unit as the downfield shifted H2, H5, and H6 protons. The anomeric region of the oligosaccharide (5.0—5.2 ppm) shows signals for the α- and β-anomers of H1 of the reducing arabinose and signals for H1 of unit *A* and *T*. These signals were distinguishable because H1 of unit *A* was split due to the long-range effect of the α- and β-anomers. Two additional signals besides the anomeric protons are present, which were assigned to the downfield shifted H2 protons of unit *A* and unit *T*. The ring protons were assigned based on COSY cross peaks. The assignments were confirmed by using the HMQC spectrum (Figure 6). Both carbons attached to the H2 protons show a ^13^C downfield shift. The δ_C_ values of these C2 carbons (84.31 and 84.57 ppm, Table 1) are in good agreement with data for an (1→5)-linked arabinose with a ferulic acid attached to *O*2 (Colquhoun et al., 1994; Bunzel et al., 2005). The δ_C_ values for the reducing arabinose unit *R* and the terminal arabinose unit *T* (except for the C2 downfield shift) are also in good agreement with literature data. Due to the low sample amount, the HMBC spectrum did not provide further information; thus, it was not possible to determine which ferulic acid is linked to which arabinose unit. However, from MS and NMR data the structure of compound 3 was suggested as shown in Figure 6.

## Discussion

The isolated oligosaccharides provide new information about the fine structure of pectins. Compound 1, which contains a side chain of *O*-β-l-arabinofuranosyl-(1→3)-arabinofuranose attached to *O*3 of the arabinan backbone, is a new structural feature of arabinans. While *O*3 substitution of the arabinan backbone is a common structural feature of arabinans, β-glycosidic linkages and oligomeric arabinose side chains are rarely described. It was already demonstrated that mung bean microsomal membranes contain a β-(1→3)-arabinopyranosyltransferase, which transfers arabinopyranose units on long linear arabinan chains, but not to small arabinose oligomers. Cardoso et al. (2002) described that arabinans in olive pomace contain α-(1→3)-linked arabinobiose side chains as well as terminal β-arabinofuranoses linked to the *O*5-position of the arabinan backbone. The authors also described that this structural feature disappeared during the ripening of the fruits, indicating an important role in cell wall development (Cardoso et al., 2007). Besides a plant physiological impact, terminal β-arabinofuranoses may also affect polysaccharide fermentation in the human large intestine. To degrade arabinans containing β-arabinofuranosyl residues, a β-arabinofuranosidase is required. Recently, such an enzyme was cloned and characterized from *Bifidobacterium longum* (Fujita et al., 2014). If other bacterial strains are not able to express this enzyme, there would be an enhancement of the prebiotic effect of the polysaccharides.

To the best of our knowledge, compound 2 is the first evidence that arabinopyranoses are incorporated into (1→4)-linked galactan backbones. Mostly, arabinose units are described in their furanose form, which are attached as side chains to the *O*3-position of the galactose backbone units (Habibi et al., 2004). Ishii et al. (2005) described the transfer of arabinopyranose to the terminal end of (1→4)-linked galactan chains by using microsomal membranes from mung bean. Huisman et al. (2001) digested soy bean galactans and analyzed them for galactan structures by MALDI-TOF MS and methylation analysis. They described terminal arabinopyranoses at the end of (1→4)-linked galactan chains and galactan oligosaccharides with internal arabinoses. Based on methylation analysis data, the arabinose was interpreted as a (1→5)-linked arabinofuranose. However, it cannot be excluded that (1→4)-linked arabinopyranose was present, because both structural features result in the same partially methylated alditol acetates in the methylation analysis. The existence of internal arabinopyranoses suggests that, in addition to the presence of an enzyme for the transfer of arabinopyranose units to galactans, there could also be an enzyme to transfer galactose units to arabinopyranoses. These structural elements may also significantly alter plant cell wall physiology and the fermentation of galactans in the human large intestine. It was demonstrated that galactans are degraded during fruit ripening, probably through plant derived enzymes such as β-galactosidase (Pena and Carpita, 2004; Goulao et al., 2007). Without enzymes hydrolyzing internal arabinopyranoses, degradation in the plant cell wall as well as in the large intestine would be limited. The required enzyme is an α-arabinopyranosidase, which was already isolated from *Bifidobacterium breve* (Shin et al., 2003).

Compound 3 demonstrates that two adjacent arabinoses can carry ferulic acid residues at position *O*2 in sugar beet arabinans. Levigne et al. (2004) isolated an arabinan oligosaccharide which also contained three arabinoses and two ferulic acids. Based on MS^2^ data, they proposed that the two non-reducing arabinoses are substituted by ferulic acid at positions *O*2 and *O*5. Because the arabinan backbone is characterized by (1→5)-linkages, this structural element is limited to chain ends of the arabinans. The structural element presented here can, however, be located within the arabinan chain. As a consequence, the enzymatic degradation of these sites by feruloyl esterase may be influenced by the high degree of substitution. Although, it was reported that feruloyl substitution does not influence the fermentation by human intestinal microbiota (Funk et al., 2007; Holck et al., 2011), single structural elements were not investigated in these studies.

This study highlights the importance of NMR spectroscopy in the structural studies of carbohydrates. Although LC-MS^2^ is a most useful tool to obtain first structural information or to confirm known structural elements, it would not have been possible to unambiguously characterize these oligosaccharides without detailed NMR studies. In addition, all three compounds cannot be specifically detected in methylation analysis, as this method cannot distinguish between (1→5)-linked arabinofuranose and (1→4)-linked arabinopyranose. Also, information about the anomeric configuration and ferulic acid substitution is lost by performing methylation analysis. The isolated oligosaccharides add complexity to the structure of pectic neutral side chains. Because their presence was only proven in one plant material yet, other plant materials need to be analyzed for these structural features. If they are demonstrated to be common structural elements of pectins, more detailed studies on their effects on plant physiology and microbial fermentation in the human gut will be required.

### Conflict of interest statement

The authors declare that the research was conducted in the absence of any commercial or financial relationships that could be construed as a potential conflict of interest.
